# Do diabetes and depressed mood affect associations between obesity and quality of life in postmenopause? Results of the KORA-F3 Augsburg population study

**DOI:** 10.1186/1477-7525-9-97

**Published:** 2011-11-04

**Authors:** Daniela A Heidelberg, Rolf Holle, Maria E Lacruz, Karl-Heinz Ladwig, Thomas von Lengerke

**Affiliations:** 1 Hannover Medical School, Medical Psychology Unit (OE 5430), Carl-Neuberg-Str. 1, 30625 Hannover, Germany; 2 Helmholtz Center Munich - German Research Center for Environmental Health, Institute of Health Economics and Health Care Management, Ingolstädter Landstr. 1, 85764 Neuherberg, Germany; 3 Helmholtz Center Munich - German Research Center for Environmental Health, Institute of Epidemiology II, Ingolstädter Landstr. 1, 85764 Neuherberg, Germany

**Keywords:** obesity, health-related quality of life, postmenopause, depressed mood, diabetes mellitus

## Abstract

**Background:**

To assess associations of obesity with health-related quality of life (HRQL) in postmenopausal women, and whether depressed mood and diabetes moderate these associations.

**Methods:**

Survey of 983 postmenopausal women aged 35-74, general population, Augsburg region/Germany, 2004/2005. Body weight/height and waist/hip circumference were assessed anthropometrically and classified via BMI ≥ 30 as obese, and WHR ≥ 0.85 as abdominally obese (vs. not). Depressed mood was assessed by the Depression and Exhaustion-(DEEX-)scale, diabetes and postmenopausal status by self-report/medication, and HRQL by the SF-12.

**Results:**

General linear models revealed negative associations of obesity and abdominal obesity with physical but not mental HRQL. Both forms of excess weight were associated with diabetes but not depressed mood. Moderation depended on the HRQL-domain in question. In non-diabetic women, depressed mood was found to amplify obesity-associated impairment in physical HRQL (mean "obese"-"non-obese" difference given depressed mood: -6.4, p < .001; among those without depressed mood: -2.5, p = .003). Reduced mental HRQL tended to be associated with obesity in diabetic women (mean "obese"-"non-obese" difference: -4.5, p = .073), independent of depressed mood. No interactions pertained to abdominal obesity.

**Conclusions:**

In postmenopausal women, depressed mood may amplify the negative impact of obesity on physical HRQL, while diabetes may be a precondition for some degree of obesity-related impairments in mental HRQL.

## Background

While the evidence on the effects of the menopausal transition on health-related quality of life (HRQL) is inconclusive [1], it is rather clear regarding effects of menopausal symptoms [2]. Avis et al. [3] found that the menopausal transition showed little impact on physical HRQL when adjusted for symptoms, medical conditions, and stress. Williams et al. [4] revealed that postmenopausal women with severe vasomotor symptoms felt more impaired in their daily activities than those with moderate or mild symptoms. Timur and Sahin [5] showed that menopause-specific quality of life was impaired in menopausal women with sleep disturbances. Finally, van Dole et al. [6] found that in postmenopausal period, increasing vasomotor symptoms were associated with a small but significant increase in psychosocial symptoms (e.g. dissatisfaction with personal life).

The role of chronic medical conditions for HRQL in postmenopause seems less clear. Avis et al. [3] studied arthritis and migraines, and found that especially the former contributed to reduced physical HRQL. Sanfélix-Genovés et al. [7] identified osteoporotic vertebral fractures to be associated with significantly lowered physical HRQL. Schwarz et al. [8] used a multi-morbidity index including hypertension, unspecified chronic back pain, arthrosis, varicosis, elevated blood lipids, migraine, thyroid disease, osteoporosis, arthritis and diabetes mellitus. Multi-morbidity was linearly associated with pain and gastrointestinal symptoms. However, due to sum-scoring no assertions could be drawn as to which diseases produced the differences. In their review, Jones and Sutton [9] argued that a condition particularly important for postmenopausal HRQL is obesity, as women tend to gain weight especially during the menopausal transition.

Although obesity has been shown to be associated with reduced physical HRQL, in most studies no association has been found for mental HRQL [10]. A comparable assertion holds for postmenopausal obesity, which is associated with poor HRQL, particularly regarding physical functioning, energy/vitality, and general health perceptions [9]. This is surprising considering the shared biology of obesity and depression [11]. Also, social stigmatisation associated with obesity may reduce mental HRQL [12]. Therefore, postmenopausal women who are obese could be expected to be more impaired in mental HRQL than their non-obese counterparts. Possibly, the existing small associations may be explained by restrictions of decreased mental HRQL to obese groups with co-morbidities. E.g., Banegas et al. [13] found cumulative effects of obesity, diabetes and hypertension on HRQL in women 60 years or older. Obese women with diabetes showed greater-than-additive declines not only in physical, but mental HRQL as well. Regarding mental morbidity, Ladwig et al. [14] found a small synergistic effect of depressed mood with obesity on long-term cardiovascular risk in obese women aged 45 to 74 years.

Considering these findings and the pathophysiological cluster including visceral fat, depressive and metabolic disorders [15], the present study investigates the synergistic effects of obesity, depressed mood and diabetes mellitus (as examples of chronic conditions) on physical and mental HRQL in postmenopausal women from the general population.

## Methods

### Population and sampling

The present sample of 983 postmenopausal women was derived as follows. To begin with, data come from a general population survey in the Augsburg region, Germany. This survey (F3) was conducted in 2004-2005 within the Cooperative Health Research in the Region of Augsburg (KORA [16]) as a follow-up to a 1994-1995 survey (S3). Central elements of data collection were a computer-aided personal interview (CAPI), a self-administered questionnaire, physical examination by trained personal (including assessments of body weight and height) and blood sampling.

The sample of the original 1994-1995 survey (S3) had been selected from 394,756 German residents aged 25-74 in 1994 via two-stage random cluster sampling. First, 17 communities were selected (probabilities proportional to size): Augsburg city and 16 communities from the two adjacent counties. In each community and within each of 10 strata defined by sex and 10-year age groups, a simple random sample was drawn from public registry office listings.

In the follow-up (F3), 3,006 S3-respondents participated (response: 76%). Additionally, of former non-responders, 178 (14%) participated, giving a total N of 3,184 (aged: 35-84). Approval of the responsible Ethics Committee (Bayerische Landesärztekammer, Munich, Germany) and informed consent of all survey participants was secured.

All participants of the follow-up F3 who were older than 74 years were excluded since some measures relevant to the present study had not been administered to them to avoid undue burden (N = 371). Underweight respondents (BMI in kg/m^2 ^<18.5, N = 15) as well as participants living outside the study region (N = 30) were excluded. Finally, 29 had refused and 7 had been too ill or had no time to fill in the questionnaire.

Of the remaining 2,732 F3-participants, all men (N = 1,312), all premenopausal women (N = 433, see below) and 4 women with no information on menopausal status were excluded from the present analysis. Thus, eventually a sample of N = 983 postmenopausal women was available for analysis.

While a non-responder analysis is not available for the KORA study F3, information on non-responders from the same population and a similar survey design can be extrapolated from a non-responder analysis of the former KORA-study S4 [17]. In this analysis, 49% of the initial non-responders had participated and - compared with responders - more often had lower education (maximally secondary school with low academic level [German: „Hauptschule"]: 65% vs. 54%) and fair or poor self-rated health (28% vs. 21%), were more often unmarried (34% vs. 29%) and smokers (29% vs. 26%), and more frequently reported physician visits in the last four weeks (46% vs. 38%), myocardial infarction (6% vs. 3%), and diabetes (7% vs. 4%).

### Measures

#### HRQL

HRQL was assessed via the first edition of German version of the SF-12 (1998, self-administered version) [18], a generic quality of life instrument with good reliability and validity. It yields one continuous summary score each for subjective physical and mental health. Scores range from 0 to 100, with higher values indicating better HRQL.

#### Postmenopausal status

Postmenopausal status was assessed via self-report in a computer-aided personal interview (CAPI) based on the items "Have you had menses within the last 12 months?", "Do you still have regular menstruation?", and "At present, are you pregnant?". Corresponding to established definitions [1], "postmenopausal" was coded given amenorrhea in the preceding 12 months and no current regular menses. Women with postmenopausal status due to surgical procedures such as oophorectomy or hysterectomy were also included. Women with systemic hormonal therapy (HT) were not automatically classified as "postmenopausal", but only if they met the indicated conditions. It was not focused on in the analyses, but considered as a confounder. HT has been argued to have the potential to improve HRQL in postmenopausal women. In this sample, current HT users (14%) reported poorer HRQL (as in [3]), especially in the mental domain. Regarding the effects of obesity, depressed mood and diabetes on HRQL, neither additional adjustment for current nor ever HT altered any of the interaction effects reported below.

#### Obesity

Obesity was assessed by anthropometric examinations. Body mass was indexed into BMI by dividing weight (kg) by squared height (m^2^). Due to subsample sizes (diabetes prevalence: 8.3%), only two BMI-groups were contrasted (WHO-classification): "non-obese" (5≤BMI<30) and "obese" (BMI ≥ 30). Abdominal obesity was defined as waist-to-hip ratio (WHR) of ≥0.85 [19]. WHR was selected since it is approximately equivalent to waist circumference regarding its association with diabetes among women [20]. Also, it is a mediator in the relationship between obesity and depression [21], and (following weight) the second most important anthropometric predictor of female bodily attractiveness [22].

#### Diabetes mellitus

Diabetes was assessed via self-report and current anti-diabetic medication. Regarding medication, participants were asked to bring drug packages or package inserts of drugs they currently use. Self-reports and medication were compared and, given conflicting data, interviewer notes and audio-recordings checked.

#### Depressed mood

Depressed mood was assessed by the Depression and Exhaustion (DEEX) scale [23] based on the von-Zerssen-Symptom-List [24]. The scale combines eight items (fatiguability, tiredness, irritability, inner tension, nervousness, anxiety, loss of energy, and difficulty in concentrating) leading to a Likert-like scale (scores from 0 to 24) normally distributed and with sufficient internal consistency (α = 0.88). Subjects in the top tertile of the distribution were considered as index group for subjects with depressed mood [23].

#### Sociodemographic/-economic variables

Gender, age and place of residence (rural vs. urban) were known via sampling. Family status and socioeconomic status (SES) were assessed via interview. SES was operationalised by school education, as in Germany it relates stronger to obesity than income or occupational status [25]. Respondents indicated their highest education level: primary or secondary general school ("Grundschule" or "Hauptschule" in Germany), intermediate secondary ("Realschule"), or grammar/upper secondary school ("Gymnasium").

### Statistical analysis

Following descriptive and bivariate analyses, general linear modelling (GLM) was conducted using the PASW-Statistics-18 software. For each HRQL summary score, one model was run to test for differences by obesity (or abdominal obesity), depressed mood, and diabetes. Because of previously reported difficulties to detect interactions in field studies [26], significance level for interactions was set at p < .1, vs. p < .05 for main effects (two-tailed). Given a significant interaction, stratified analyses were conducted to clarify the underlying pattern, i.e., with either obesity or abdominal obesity defined as the focal independent variable, simple effects or (given three-way interactions) simple simple effects [27] were tested. For stratified analysis, 95%-confidence intervals for mean differences were calculated. Outlier trimming was not applied. All models were adjusted for age, education, family status, type of health insurance (statutory vs. private), and place of residence (urban vs. rural).

## Results

### Descriptive and bivariate analysis

Table 1 describes the sample. Overall, 29.5% of the 983 women were classified as obese, while 43.1% as having abdominal obesity. Only a small minority was younger than 45 years (4.4%). Almost two thirds had only low school education. Nearly three-fourths lived with a partner. About one eighth had private health insurance, which is close to the overall German rate (10.5%). Furthermore, 44.9% lived in the city of Augsburg, 8.3% had diabetes, and 40% met the criteria for depressed mood.

**Table 1:** Sample description: bivariate cross-tabulations of demographics, diabetes mellitus and depressed mood with obesity and abdominal obesity

	Total		Non-obesity (BMI < 30) (N = 693; 70.5%)		Obesity (BMI ≥ 30) (N = 290; 29.5%)				No abdominal obesity (WHR < 0.85) (N = 559; 56.9%)		Abdominal obesity (WHR ≥ 0.85) (N = 424; 43.1%)			
Characteristic	n	%	n	%	n	%	χ^2^	P	n	%	n	%	χ^2^	p
Age (in years)														
35-44	43	4.4	34	4.9	9	3.1	19.2	< .001	34	6.1	9	2.1	52.0	< .001
45-54	238	24.2	192	27.7	46	15.9			171	30.6	67	15.8		
55-64	374	38.0	253	36.5	121	41.7			209	37.4	165	38.9		
65-74	328	33.4	214	30.9	114	39.3			145	25.9	183	43.2		
School education														
High (grammar school [Gymnasium])	102	10.4	90	13.0	12	4.2	40.8	< .001	77	13.8	25	5.9	38.1	< .001
Medium (intermediate school [Realschule])	232	23.7	188	27.2	44	15.3			157	28.2	75	17.7		
Low (maximally secondary general school [Hauptschule])	645	65.9	413	59.8	232	80.6			322	57.9	323	76.4		
Family status														
Living with partner	712	72.5	501	72.4	211	72.8	0.0	< .908	408	73.0	304	71.9	0.2	.697
Not living with partner	270	27.5	191	27.6	79	27.2			151	27.0	119	28.1		
Health insurance														
Private	124	12.6	89	12.8	35	12.1	0.1	.739	75	13.4	49	11.6	0.8	.384
Statutory	859	87.4	604	87.2	255	87.9			484	86.6	375	88.4		
Place of residence														
Rural	542	55.1	381	55.0	161	55.5	0.0	.877	315	56.4	227	53.5	0.8	.380
Urban	441	44.9	312	45.0	129	44.5			244	43.6	197	46.5		
Depressed mood (DEEX-scale)														
Yes	392	40.0	272	39.4	120	41.7	0.5	.503	224	40.1	168	40	0.0	.982
No	587	60.0	419	60.6	168	58.3			335	59.9	252	60		
Diabetes mellitus														
Yes	82	8.3	37	5.3	45	15.5	27.7	< .001	20	3.6	62	14.6	38.5	< .001
No	901	91.7	656	94.7	245	84.5			539	96.4	362	85.4		

Bivariately, women with both general and abdominal obesity were older than their non-obese counterparts, and more often had completed secondary general school only. Regarding diabetes, its prevalence was about threefold in the obese group, and about fourfold in those with abdominal obesity (15.5% vs. 5.3% and 14.6% vs. 3.6%, respectively).

Table 2 shows the mean scores of the SF-12 in different subgroups. Physical HRQL decreases with age, lower school education, and is lower in participants with statutory health insurance. Additionally, it is significantly lower in participants with obesity, abdominal obesity, depressed mood, and diabetes. In contrast, mental HRQL is significantly lower only in participants with depressed mood, and marginally decreased in those not living with a partner and those with statutory health insurance.

**Table 2:** Physical and mental health-related quality of life (SF-12) in different sub-groups: unadjusted bivariate analysis

		SF-12 Physical Sum Score			SF-12 Mental Sum Score		
Source of variation		Mean	95%-CI		Mean	95%-CI	
Age (in years)	35-44	48.7	45.9-51.5	F_(3,828) _= 6.8, p ≤ .001	50.0	46.8-53.1	F_(3,828) _= 1.5, p = .206
	45-54	48.2	47.0-49.5		48.8	47.5-50.2	
	55-64	46.2	45.2-47.2		56.1	49.0-51.2	
	65-74	44.7	43.6-45.8		50.8	49.6-52.0	
Education	High (grammar school)	49.5	47.6-51.5	F_(2,825) _= 5.6, p = .004	49.4	47.2-51.6	F_(2,825) _= 0.6, p = .557
	Medium (intermediate school)	46.2	44.9-47.4		49.5	48.2-50.9	
	Low (max. secondary general school)	46.0	45.2-46.7		50.3	49.4-51.2	
Family status	Living with partner	46.7	45.9-47.4	F_(1,829) _= 2.6, p = .110	50.4	49.6-51.2	F_(1,829) _= 3.7, p = .056
	Not living with partner	45.5	44.3-46.7		48.9	47.5-50.2	
Health insurance	Private	48.1	46.3-49.8	F_(1,830) _= 4.2, p = .041	51.7	49.8-53.6	F_(1,830) _= 3.5, p = .061
	Statutory	46.1	45.4-46.8		49.7	49.0-50.5	
Place of residence	Rural	46.7	45.9-47.5	F_(1,830) _= 1.5, p = .223	49.6	48.7-50.5	F_(1,830) _= 1.4, p = .232
	Urban	45.9	44.9-46.9		50.5	49.4-51.5	
Obesity (BMI ≥ 30)	Yes	43.0	41.8-44.1	F_(1,830) _= 47.3, p ≤ .001	50.2	48.9-51.5	F_(1,830) _= 0.1, p = .759
	No	47.7	47.0-48.5		50.0	49.1-50.7	
Abdominal Obesity (WHR ≥ 0.85)	Yes	45.4	44.4-46.4	F_(1,830) _= 6.1, p = .014	49.9	48.8-51.0	F_(1,830) _= 0.1, p = .823
	No	47.0	46.2-47.9		50.1	49.2-51.0	
Depressed mood (DEEX-scale)	Yes	42.5	41.6-43.4	F_(1,829) _= 108.9, p ≤ .001	43.3	42.3-44.2	F_(1,829) _= 341.8, p ≤ .001
	No	49.0	48.2-49.7		54.5	53.7-55.2	
Diabetes mellitus	Yes	42.4	40.1-44.6	F_(1,830) _= 13.1, p ≤ .001	48.5	46.0-51.0	F_(1,830) _= 1.6, p = .213
	No	46.7	46.1-47.4		50.1	49.4-50.8	

### GLM

In the four GLM, the hypotheses that the relations between obesity defined by BMI or WHR and HRQL are moderated by depressed mood and diabetes were scrutinized. Table 3 shows the results for obesity (BMI ≥ 30) and Table 4 for abdominal obesity (WHR ≥ 0.85) both for the physical sum score (left column) and the mental sum score (right column) of the SF-12, respectively. Regarding physical HRQL (SF-12 Physical Sum Score), for which results will be described first, all main effects (obesity, depression, and diabetes) as well as the three-way interaction are significant in the model with BMI (Table 3, left column). As the adjusted means for the main effects show, physical HRQL is lower in the presence of obesity, depressed mood, or diabetes, respectively (the pattern underlying the significant three-way interaction will be described in the next paragraph). In contrast, only the main effects of depressed mood and diabetes (and thus no interactions) are significant in the model with WHR (Table 4, left column). While here, the association of abdominal obesity with impaired physical HRQL in bivariate analysis is attenuated, adjusted means show that physical HRQL is impaired given either depressed mood or diabetes mellitus. This indicates that the association of abdominal obesity is mediated by one or both of depressed mood or diabetes mellitus as given co-morbidities.

**Table 3:** Physical and mental HRQL (SF-12) by obesity, diabetes mellitus, and depressed mood: GLM results^a^

		SF-12 Physical Sum Score			SF-12 Mental Sum Score		
Source of variation	Statistic	Value	95%-CI	Effect	Value	95%-CI	Effect
Obesity (BMI ≥ 30)							
Yes	Adjusted mean	42.0	40.5-43.6	F_(1,825) _= 7.7, p = .006	47.7	46.2-49.3	F_(1,825) _= 0.3, p = .588
No	Adjusted mean	45.2	43.6-46.8		48.3	46.7-49.9	
Depressed mood (DEEX-scale)							
Yes	Adjusted mean	39.7	38.0-41.5	F_(1,825) _= 46.5, p < .001	41.7	39.9-43.5	F_(1,825) _= 122.4, p < .001
No	Adjusted mean	47.5	46.0-48.9		54.4	52.9-55.8	
Diabetes mellitus							
Yes	Adjusted mean	42.2	40.0-44.3	F_(1,825) _= 6.4, p = .012	46.7	44.5-48.9	F_(1,825) _= 5.3, p = .022
No	Adjusted mean	45.0	44.4-45.7		49.4	48.7-50.1	
Obesity × Depressed Mood ^b^				F_(1,825) _= 0.1, p = .775			F_(1,825) _= 2.7, p = .104
Obesity × Diabetes mellitus ^b^				F_(1,825) _= 1.4, p = .236			F_(1,825) _= 3.2, p = .074
Depressed Mood × Diabetes mellitus ^b^				F_(1,825) _= 0.6, p = .431			F_(1,825) _= 3.7, p = .053
Obesity × Depressed Mood × Diabetes mellitus ^b^				F_(1,825) _= 4.1, p = .044			F_(1,825) _= 0.6, p = .447

Notes: ^a ^Adjusted for age, school education, family status, type of health insurance (statutory vs. private), and place of residence (urban vs. rural) ^b ^To simplify presentation, adjusted means for subgroups are not shown here (see below, interaction contrast analyses in figures 1 to 3)

**Table 4:** Physical and mental HRQL (SF-12) by abdominal obesity, diabetes mellitus, and depressed mood: GLM results^a^

		SF-12 Physical Sum Score			SF-12 Mental Sum Score		
Source of variation	Statistic	Value	95%-C	Effect	Value	95%-CI	Effect
Abdominal Obesity (WHR ≥ 0.85)							
Yes	Adjusted mean	44.0	42.7-45.4	F_(1,825) _= 0.0, p = .978	47.7	46.4-49.1	F_(1,825) _= 0.0, p = .990
No	Adjusted mean	44.1	42.0-46.2		47.8	45.7-49.9	
Depressed mood (DEEX-scale)							
Yes	Adjusted mean	40.2	38.3-42.2	F_(1,825) _= 35.9, p < .001	40.9	38.9-42.8	F_(1,825) _= 118.0, p < .001
No	Adjusted mean	47.9	46.3-49.5		54.6	53.0-56.2	
Diabetes mellitus							
Yes	Adjusted mean	42.2	39.7-44.6	F_(1,825) _= 8.6, p = .003	46.5	44.1-48.9	F_(1,825) _= 3.8, p = .051
No	Adjusted mean	46.0	45.0-46.6		49.0	48.4-49.6	
Abdominal Obesity × Depressed Mood ^b^				F_(1,825) _= 0.4, p = .834			F_(1,825) _= 2.1, p = .144
Abdominal Obesity × Diabetes mellitus ^b^				F_(1,825) _= 0.3, p = .583			F_(1,825) _= 0.2, p = .670
Depressed Mood × Diabetes mellitus ^b^				F_(1,825) _= 1.2, p = .270			F_(1,825) _= 5.2, p = .022
Abdominal Obesity × Depressed Mood × Diabetes mellitus ^b^				F_(1,825) _= 0.6, p = .444			F_(1,825) _= 2.4, p = .125

Notes: ^a ^Adjusted for age, school education, family status, type of health insurance (statutory vs. private), and place of residence (urban vs. rural) ^b ^To simplify presentation, adjusted means for subgroups are not shown here (see below, interaction contrast analyses in figures 1 to 3)

**Figure 1:**
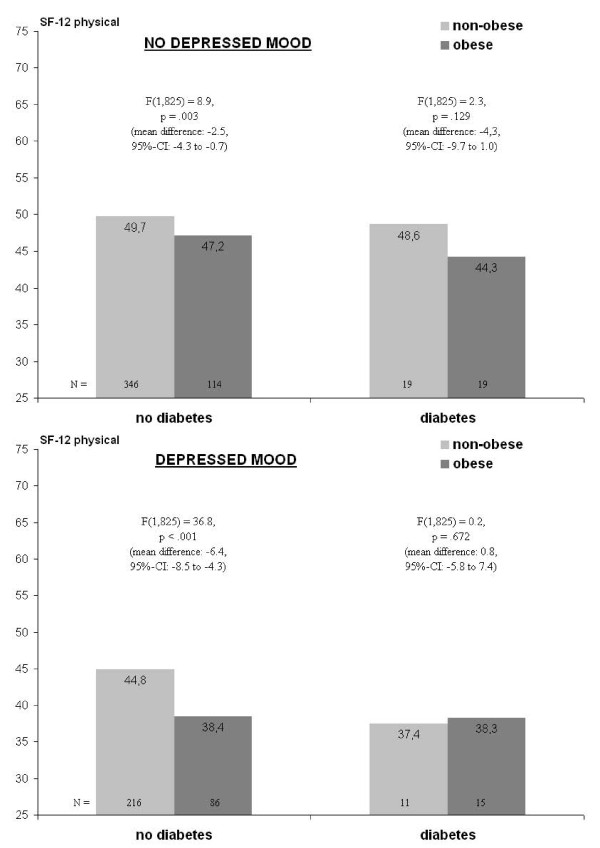
**Three-way interaction of obesity, diabetes and depressed mood on physical HRQL (SF-12)^a,b^**. ^a ^adjusted for age, education, family status, type of health insurance, and place of residence (urban vs. rural). ^b ^F-values represent simple simple effects of obesity within the combinations of depressed mood and diabetes.

Figure 1 shows the pattern underlying the three-way interaction in the model with obesity reported in Table 3 (F = 4.1, p = .044). Obesity is significantly associated with lower levels of physical HRQL only among non-diabetic women irrespective of depressed mood. Though among non-depressed diabetic women, the difference between obese vs. non-obese is numerically larger, it is statistically insignificant. Simultaneously, among those with both depressed mood and diabetes, the difference between obese and non-obese women is smallest among all comparisons.

Further exploration of the three-way interaction (not shown) revealed that while the two-way interaction of obesity with diabetes was significant both among those with and without depressed mood (F = 27.6 and F = 6.7, both p ≤ .01), the two-way interaction of obesity with depressed mood was significant only in the group without but not in that with diabetes (F = 7.4, p = .007 vs. F = 0.7, p = .405). In other words, in non-diabetic participants, the effect from BMI on physical HRQL is significantly amplified given depressed mood, i.e. the mean difference of -6.4 shown in Figure 1 is significantly higher than the mean difference of -2.5.

**Figure 2:**
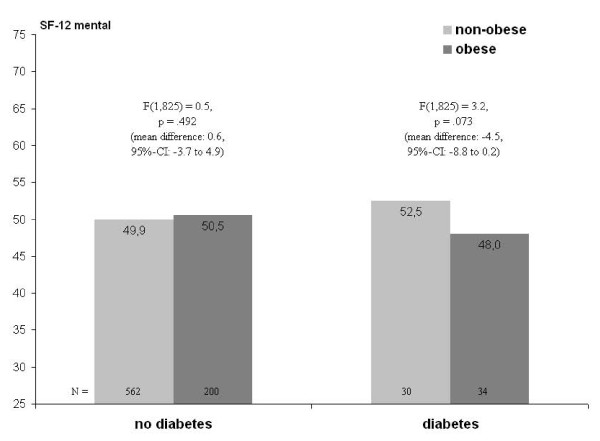
**Two-way interaction of obesity and diabetes on mental HRQL (SF-12)^a,b^**. ^a ^adjusted for age, education, family status, type of health insurance, and place of residence (urban vs. rural). ^b ^F-values represent simple effects of obesity within groups defined by diabetes status.

**Figure 3:**
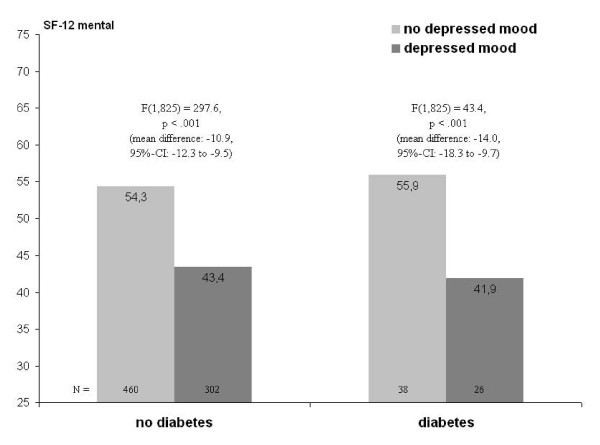
**Two-way interaction of depressed mood and diabetes on mental HRQL (SF-12)^a,b^**. ^a ^adjusted for age, education, family status, type of health insurance, and place of residence (urban vs. rural). ^b ^F-values represent simple effects of depressed mood within groups defined by diabetes status.

Turning to mental HRQL, main effects of depressed mood and diabetes are seen, with the effect of depressed mood being considerably stronger (Table 3 and Table 4, right columns). In contrast, neither obesity nor abdominal obesity is significantly related to mental HRQL. While in both models the interaction between depressed mood and diabetes is significant, in the model with obesity the interaction with diabetes is significant as well. Figure 2 and Figure 3 show the underlying patterns. On one hand, obesity is associated with a marginally significant lower level of mental HRQL among women with diabetes, with no difference among those without diabetes (see Figure 2). On the other hand, depressed mood is associated with lower mental HRQL regardless of diabetes status, however more strongly so in the diabetes group (see Figure 3; estimates are from the BMI-model and equivalent to the WHR-model).

## Discussion

Negative associations with physical but not mental HRQL were found for general and abdominal obesity in a community sample of postmenopausal women. Both obesity-indicators were associated with diabetes but not depressed mood, the latter being in line with cross-sectional studies from populations other than the US finding no associations between obesity and depression [28]. Moderating effects of depressed mood and diabetes on the relation between obesity and HRQL depended on the HRQL-dimension. Depressed mood significantly reduced the score in physical HRQL, given no diabetes. In contrast, reduced mental HRQL associated with obesity was restricted to women with diabetes (independent of depressed mood). Finally, the effect of depressed mood in terms of reduced mental HRQL was found both in diabetic and non-diabetic women, but was stronger in the former group. Interactions between abdominal obesity and depression or diabetes were not observed.

These findings add observational evidence to the field of postmenopause, HRQL, and chronic medical conditions. In particular, that depressed mood as a mental ill-health state amplifies the negative impact of obesity on physical HRQL (given a healthy state in terms of no diabetes), while diabetes (as a physical disease) turned out to be a precondition for obesity-related impairments in mental HRQL, reflects complex interrelations. Also, it is intriguing that these patterns were found for general but not abdominal obesity, especially given the latter's significant role in the postmenopausal period [29]. Myint et al. [30] found that an increase in WHR, but not in BMI, was significantly associated with lower mental health. The present finding that an elevated BMI was associated with lower mental HRQL in diabetic participants may reflect that general obesity as a stressor may potentiate its unfavourable effect on mental HRQL when combined with a chronic condition.

Moreover, it is notable that the three-way interaction between obesity, depressed mood and diabetes regarding physical HRQL was driven more by the interaction of obesity with depressed mood than with diabetes. "Depressed mood" as defined by the DEEX-scale differs from other measures as it detects physical, non-stigmatizing symptoms, and resembles the concept of vital exhaustion [31]. This "general malaise" might prevent coping with the strains obesity imposes on physical HRQL. In contrast, diabetes may not only moderate, but also mediate the association between obesity and physical HRQL (similar to abdominal obesity), not least because the etiological role of (abdominal) obesity for diabetes is more clear-cut than for depressed mood [13,14,20,21,28].

### Strengths and limitations

A major strength of this study is the rigorous quality assurance applied during data collection [16], allowing to analyse a definite postmenopausal cohort with standardized, validated instruments. First, a limitation that derives from the observational, cross-sectional design is that reversed or bidirectional causality could not be ruled out. However, effects of chronic conditions on the relation between obesity and HRQL in postmenopausal women have hardly been studied, warranting report of the results.

Second, both the absolute sample size and observational approach implied an unbalanced design, of which subsample sizes are indicative. While generally, small subsamples tend to work against detecting significant differences (thus testing conservatively), more sophisticated analyses were unfeasible. Only two BMI- and WHR-groups along could be differentiated (e.g., there were only three normal weight women with diabetes). Similarly, different diabetes types could not be contrasted since only one of 82 had type 1 diabetes. Thus, results by and large reflect effects of type 2 diabetes. Also, factors such as other concomitant diseases, parity or sexual activity could not be considered. The choice of the DEEX-scale [23] in order to operationalise depressed mood was influenced by subsample sizes as well. This instrument has been shown to be useful to identify depressed mood in otherwise apparently healthy subjects in general populations. At the same time, unlike the Hospital Anxiety and Depression Scale it is not specifically designed for groups with physical diseases, and unlike the Patient Health Questionnaire-9 not directly based on the diagnostic criteria for major depressive disorders. However, in the present survey using these alternatives would have resulted in one-digit subsample sizes not suitable for analysis.

Third, the response rate (76%), though comparing well to surveys with comparable participation time (in the present survey this was, on average, 175 minutes, which include all parts of the survey performed at one visit at the study centre, and possible breaks during this visit), may lead to selection biases, as healthier subjects are more likely to participate. Indeed, a non-responder survey in another KORA-study (S4) has revealed that responders tend to be healthier (e.g. in terms of lower diabetes rates; for details, see "Population and sampling"). Yet, this rather reduces ability to detect associations.

Fourth, HRQL-assessment by the SF-12 implies restrictions. Unlike the SF-36 it does not allow to analyse sub-dimensions of physical and mental HRQL (regarding its sum scores, however, it does compare well to the SF-36 in the context of obesity [32]]. Also, the SF-12 is a generic instrument, and might not reflect menopause-specific HRQL-dimensions as would e.g. the Menopause-specific Quality of Life Questionnaire [33] or the Menopause Rating Scale [34]. While the choice of the SF-12 related to the fact that the KORA-survey was not specifically designed to study menopausal issues, using a generic instrument may also have advantages in a study which scrutinizes different conditions (obesity, diabetes, and depressed mood) as joint determinants of postmenopausal HRQL. Thus, using a condition-specific instrument might have overlooked HRQL-effects of other conditions, respectively. Also, generic mental health-related quality of life has been shown to be affected by the greatest reductions after weight gain in a recent trial which included an obesity-specific measure (Impact of Weight on Quality of Life-Lite) [35].

Fifth, variances accounted for in GLM were 19% for physical and 31% for mental HRQL in the models with obesity, and 14% and 30% in those with abdominal obesity. Those explained by significant interactions did not exceed one percent. Also, cross-validating the complex interactions e.g. by partitioning was not possible, again due to sample size restrictions. In terms of clinical significance, however, the HRQL-impairments identified are important. Subgroups reporting poorest physical HRQL (obesity/depressed mood, and depressed mood/diabetes) were worse off than those with either diabetes or any cancer (excluding skin carcinoma) in the SF-12 normative sample [18]. This also holds for the obesity-associated impairment in mental HRQL among women with diabetes.

## Conclusions

This study provides observational evidence that depressed mood significantly elevates obesity-associated impairment in physical HRQL in postmenopausal women in absence of a chronic condition (here: diabetes), and that a significant reduction in *mental *HRQL is restricted to obese women with diabetes. These effects were not observed for abdominal obesity. By joint scrutiny of different chronic conditions, this study follows the call to consider clusters of symptoms, and mechanisms common to the clusters, in the context of developing a theoretical model of menopause, its symptoms, and quality of life [36]. It may contribute to tailoring interventions fostering HRQL in postmenopausal women. Regarding physical HRQL, women most in need may be those obese and feeling depressed. Regarding mental HRQL, obesity and diabetes as interacting factors seem worth of further scrutiny. In future studies, the underlying pathophysiological mechanisms should be investigated. Finally, lifestyle interventions should take into account low HRQL associated with concomitant depressed mood and diabetes, as it is a pre-treatment predictor of unsuccessful weight control [37].

## List of abbreviations

BMI: body mass index; GLM: General Linear Models; HRQL: health-related quality of life; WHR: waist-to-hip ratio.

## Competing interests

The authors declare that they have no competing interests.

## Authors' contributions

DAH participated in the statistical analyses and the writing of the article. RH participated in the preparation and conduct of the study and the editing of the article. MEL participated in the conduct of the study and the editing of the article. KHL participated in the preparation and conduct of the study and the editing of the article. TvL participated in the preparation and conduct of the study, the statistical analyses and the writing of the article. All authors read and approved the final manuscript.
